# MST4 inhibits human hepatocellular carcinoma cell proliferation and induces cell cycle arrest via suppression of PI3K/AKT pathway

**DOI:** 10.7150/jca.45822

**Published:** 2020-06-28

**Authors:** Wei-Chao Hao, Qiu-Ling Zhong, Wen-Qian Pang, Mei-Juan Dian, Jing Li, Liu-Xin Han, Wen-Tao Zhao, Xiao-Ling Zhang, Sheng-Jun Xiao, Dong Xiao, Xiao-Lin Lin, Jun-Shuang Jia

**Affiliations:** 1 Guangdong Provincial Key Laboratory of Cancer Immunotherapy Research and Guangzhou Key Laboratory of Tumor Immunology Research, Cancer Research Institute, School of Basic Medical Sciences, Southern Medical University, Guangzhou 510515, China.; 2 Institute of Comparative Medicine & Laboratory Animal Center, Southern Medical University, Guangzhou 510515, China.; 3 Radiotherapy Center, the First People's Hospital of Chenzhou, Chenzhou 423000, China.; 4 The third people's hospital of Kunming, Kunming 650041, China.; 5 Department of Medical Oncology, The Third Affiliated Hospital of Kunming Medical University (Tumour Hospital of Yunnan Province), Kunming 650118, China.; 6 Department of Physiology, Faculty of Basic Medical Sciences, Guilin Medical University, Guilin 541004, China.; 7 Department of Pathology, the Second Affiliated Hospital, Guilin Medical University, Guilin 541199, China.

**Keywords:** MST4, Hepatocellular carcinoma (HCC), Proliferation, Cell cycle, PI3K/AKT pathway

## Abstract

**Objective:** MST4 has exhibited functions in regulating cell polarity, Golgi apparatus, cell migration, and cancer. Mechanistically, it affects the activity of p-ERK, Hippo-YAP pathway and autophagy. The aim of this study is to further examine the functions of MST4 in hepatocellular carcinoma (HCC) and the underlying mechanism.

**Methods:** The expression level of MST4 in HCC and noncancer adjacent liver tissues was determined by qRT-PCR and immunohistochemistry staining. Wild-type MST4 (MST4) and a dominant-negative mutant of MST4 (dnMST4) were overexpressed in HCC cell lines, respectively. CCK-8 assay, EdU incorporation assay, and soft agar assay were used to determine cell proliferation *in vitro*. The xenograft mouse model was employed to determine HCC cell growth *in vivo*. Cell cycle analysis was performed by PI staining and flow cytometry. The expression of key members in PI3K/AKT pathway was detected by Western blot analysis.

**Results:** In our study, we reported new evidence that MST4 was frequently down-regulated in HCC tissues. Gain-of-function and loss-of-function experiments demonstrated that MST4 negatively regulated *in vitro* HCC cell proliferation. Additionally, MST4 overexpression suppressed Bel-7404 cell tumor growth in nude mice. Further experiments revealed that the growth-inhibitory effect of MST4 overexpression was partly due to a G1-phase cell cycle arrest. Importantly, mechanistic investigations suggested that dnMST4 significantly elevated the phosphorylation levels of key members of PI3K/AKT pathway, and the selective PI3K inhibitor LY294002 can reverse the proliferation-promoting effect of dnMST4.

**Conclusions:** Overall, our results provide a new insight into the clinical significance, functions and molecular mechanism of MST4 in HCC, suggesting that MST4 might have a potential therapeutic value in the HCC clinical treatment.

## Introduction

Hepatocellular carcinoma (HCC) accounts for the vast majority of primary liver cancer, which has the sixth-highest incidence among cancers worldwide and is the fourth leading cause of cancer-related death 1. Although the etiology and risk factors of HCC have been clearly studied, its diagnosis and treatment are still challenging. Due to asymptomatic disease progression of HCC, most patients are diagnosed at an advanced stage. Surgical resection is currently the most effective treatment of HCC, but it is only suitable for about 20% of patients, and tumor recurrence is very common 2. Systematic chemotherapy, such as molecular targeted drugs sorafenib and lenvatinib, is recommended for inoperable patients with advanced HCC, but they can only extend the survival time of patients by only three months 3, 4. Nivolumab, a PD-1 immune checkpoint inhibitor, achieved a median survival time of 15.6 months in a single-group phase 2 trial. However, the overall response was only 14.3% according to Response Evaluation Criteria in Solid Tumors (RECIST) 5-7. Therefore, due to the limited options for diagnosis and therapies of HCC, understanding the molecular mechanisms for HCC carcinogenesis and development is very essential.

Mammalian sterile-20-like kinase 4 (MST4) is a member of the germinal center kinase (GCK) group III family of kinases, which is a subset of the Ste20-like kinases 8. Previous studies have reported the role of MST4 in a variety of biological processes. For example, MST4 can be located at the Golgi apparatus by binding to the Golgi matrix protein GM130, thus regulating the morphology of Golgi and controlling cell migration 9. In intestinal cells, MST4 can mediate the LKB1 control of cell polarity by phosphorylating ezrin at T567 10. Additionally, a recent study indicated MST4 could form a complex with MOB4 to regulate MST1 thus affecting Hippo-YAP signaling 11. Meanwhile, some studies have also revealed the important role of MST4 in cancers. For instance, STRIPAK complex was demonstrated to regulate breast cancer cell migration and metastasis by controlling MST4 activity 12. In gastric cancer, MST4 is reported to facilitate p-ERK pathway thus promoting epithelial-mesenchymal transition (EMT) and metastasis 13. In glioblastoma, the expression of MST4 is related to the induction of tumor autophagy and can lead to tumorigenesis 14. However, its actual role and underlying molecular mechanisms in diverse malignancies are still not fully understood.

In our study, we observed that MST4 expression is frequently down-regulated in HCC tissues compared with corresponding noncancer adjacent liver tissues. Overexpression of MST4 remarkably suppressed HCC cell proliferation *in vitro* and *in vivo*, and vice versa. Additionally, our studies showed that MST4 can inhibit the phosphorylation of AKT and GSK-3β, which leads to the G1 phase cell cycle arrest. Importantly, our study demonstrated, for the first time, that MST4 can suppress HCC cell proliferation and cell cycle progression by inactivating PI3K/AKT pathway, providing new insight into the role and molecular mechanism of MST4 in cancer.

## Materials and Methods

### Cell lines and cell culture

Human HCC cell lines (i.e. Bel-7402 and Bel-7404) were obtained from the Cell Bank of Shanghai Institutes for Biological Sciences (SIBS). All cell lines were cultured in Dulbecco's modified Eagle medium (DMEM, Corning, USA) supplemented with 10% fetal bovine serum (FBS, PAN, Germany) in a humidified incubator with 5% CO2 at 37°C. Bel-7402 and Bel-7404 cells were subjected to the gain-of-function and loss-of-function experiments. The proliferation rate of Bel-7404 cells is higher than that of Bel-7402 cells.

### Tissue specimens

66 paired specimens of primary HCC tissues and adjacent non-cancerous liver tissues (for qRT-PCR) were freshly collected from Nanfang Hospital, Southern Medical University (Guangzhou, China) with informed consent and under institutional review board-approved protocols. All fresh samples were frozen immediately in liquid nitrogen. Total RNAs were extracted from the samples and analyzed by qRT-PCR. 105 pairs formalin-fixed paraffin-embedded HCC and adjacent non-cancerous liver biopsies were obtained from the Department of Pathology, the Second Affiliated Hospital of Guilin Medical College, China, between 2009 and 2013. All clinical HCC specimens used in this study were histopathologically and clinically diagnosed. None of the HCC patients received preoperative radiotherapy or chemotherapy. Retrieval of tissue and clinical data was performed according to the regulations of the institutional review boards (IRB) of the Second Affiliated Hospital of Guilin Medical University and data safety laws with specific regard to ethical standards and patient confidentiality. Written informed consent was signed by each patient. Ethical approval was given by the Medical Ethics Committee of Southern Medical University. The study is compliant with all relevant ethical regulations involving human participants.

### Histological analysis and immunohistochemistry (IHC)

Histological analysis and immunohistochemical staining were performed as described previously 15, 16. Briefly, after deparaffinization, rehydration and antigen retrieval, tissue slides (4μm thick) were blocked with 3% H_2_O_2_ for 10 min and incubated with primary antibodies, including rabbit anti-MST4 (Abcam; ab52491) and mouse anti-BrdU(GE Healthcare; RPN202), at 4°C overnight. The slides were then stained with a corresponding secondary antibody at 37°C for 20 min. Finally, the sections were stained with diaminobenzidine (DAB) and counterstained with hematoxylin. Histopathology and immunohistochemistry analysis were performed independently by two pathologists without knowledge of the backgrounds of the patients. Staining intensity and percentage of staining-positive cells were evaluated for the IHC scores. Staining intensity was classified as 0 (negative), 1 (weak), 2 (moderate) and 3 (strong). Percentage of stained cells were classified as 0 (<5 %), 1 (≥5%, <25 %), 2 (≥25, <75%) and 3 (≥75%). The final IHC score is the score of intensity multiplied by the score of percentage. The scores 0, 1, 2, 3, 4 were defined as low, and the scores 6 and 9 were defined as high. Slides with conflicting evaluations were reassessed, and a consensus was reached.

### RNA isolation, reverse transcription, and quantitative real-time PCR (qRT-PCR)

Total RNAs were extracted using RNAiso Plus (Takara, China). cDNA was synthesized using the PrimeScript™ RT Reagent Kit (Takara, China) according to the manufacturer's protocol. qRT-PCR was performed with SYBR® Premix Ex Taq™ Kit (Takara, China). The following primers were used: MST4, forward 5'-TTCGAGCTGGTCCATTTGATG-3' and reverse 5'-TGAATGCAGATAGTCCAGACCT-3'; GAPDH (endogenous control), forward 5'- ACCCAGAAGACTGTGGATGG-3' and reverse 5'- TCTAGACGGCAGGTCAGGTC-3'. The fold changes of mRNA expression were detected on a LightCycler 96 instrument (Roche) through the 2-ΔΔCt method 17.

### Plasmids

The pcDNA3.1-MST4-WT and pcDNA3.1-MST4-T178A plasmids were kindly provided by Prof. Hans Clevers^10^. The wild-type MST4-expressing lentiviral vector (pLV-MST4) was prepared by subcloning the human MST4 coding sequence from pcDNA3.1-MST4-WT 10 into the lentivirus vector. The dominant-negative mutant MST4-expressing lentiviral vector (pLV-dnMST4) was prepared by subcloning the mutant human MST4 sequence from pcDNA3.1-MST4-T178A 10 into the lentiviral vector. The lentiviral packaging plasmids psPAX2 and pMD2.G were kindly provided by Prof. Didier Trono (University of Geneva, Geneva, Switzerland).

### Viral preparation and transduction

The LV-MST4, pLV-dnMST4, or the pCDH-EF1-GFP empty vector was respectively transfected into 293T cells, with the lipofectamine 2000 (Invitrogen, USA) and lentivirus packaging plasmids psPAX2 and pMD2G. After being cultured for 72 hours, the supernatant was harvested and the lentivirus was concentrated. For transduction, cells were seeded in petri dishes and incubated with lentivirus and 10 ug/mL polybrene (Sigma, USA) for 8 hours.

### Western blot

The cell lysates were separated by 10% SDS-PAGE gels and electrophoretically transferred to PVDF membranes (Millipore, USA). The membranes were then incubated with primary antibodies, including rabbit anti-MST4(Abcam; ab52491), rabbit anti-p-AKT(S473)(CST; 4060S), rabbit anti-AKT(CST; 4685S), rabbit anti-GSK3 β (CST; 9323S), rabbit anti-p-GSK3 β (CST; 9315S) and rabbit anti-GAPDH (PTG; 10494-1-AP), overnight at 4°C, followed by incubation with an HRP-labeled goat anti-rabbit IgG for 1 h at room temperature. The detections were carried out on a chemiluminescence imaging system (Bio-Rad, USA). GAPDH was used as an internal control.

### Immunofluorescence (IF) staining

IF staining was performed as previously described 18 using the antibody rabbit anti-MST4 (Abcam; ab52491). The slides were counterstained with DAPI (Sigma) to visualize the nuclei and imaged with a fluorescence microscope (Nikon, Japan).

### CCK-8 assay

Cells were seeded in 96-well plates with 1000 cells per well. The cell growth rates were assessed by Cell Counting Kit-8 (Dojindo; CK04) for the indicated time. The plates were incubated at 37°C for an additional 2 hours after CCK-8 treatment. Absorbance at 450nm was read by a microplate reader (BioTek, USA) and recorded.

### EdU incorporation assay

The proliferating cells were examined using Cell-Light™ EdU Apollo567 *In vitro* Kit (C10310-1; RiboBio, Guangzhou, China) according to the manufacturer's protocol. Briefly, cells were seeded in 24-well plates for 24 hours with fresh medium and cultured with 50 μM EdU for an additional 2 hours, then fixed by 4% paraformaldehyde for 30 minutes. After being washed twice with PBS containing 0.5% Triton X-100, cells were treated with the reaction dye for an additional 30 minutes while shielded from light. Finally, cells were counterstained with Hoechst 33342. The images were collected under a fluorescence microscope (Nikon, Japan) with 40X visions.

### Soft agar assay

This assay was performed to determine the anchorage-independent growth ability of cells. Briefly, 1ml 0.6% bottom agar per well were plated within 6-well plates first. Then 1×10^4^ cells seeded in 1ml 0.3% top agar were covered on the bottom agar of each well, and incubated in a humidified incubator with 5% CO^2^ at 37°C for 2-3 weeks. Colonies were photographed with 40X visions under a microscope (Nikon, Japan) and counted.

### Cell cycle analysis

Cells were harvested and washed with cold PBS, and then fixed with 70% ice-cold ethanol at -20°C overnight. After centrifugation, cells were stained with propidium iodide (PI) and RNase for 30 minutes shielded from light at room temperature. Cell cycle distribution was then analyzed on a BD FACSCalibur. 1×10^4^ cells were measured for each sample.

### Hypoxia assay

The cancer cell hypoxia model was created based upon the hypoxic microenvironment (5% O_2_). In this study, the Hypoxia Incubator Chamber (Cat#27310; STEMCELL TECHNOLOGIES) for generation of a hypoxic environment for cell is a self-contained and sealed chamber that fits inside existing laboratory incubators. The chambers have an integrated stacking feature for storage during or after experimentation.

### *In vivo* tumorigenicity assay

BALB/c nude mice aged 4 to 5 weeks were purchased from the Medical Laboratory Animal Center of Guangdong Province. Vector- or MST4-expressing cancer cells (2×10^6^ cells for Bel-7404 cells) were subcutaneously injected into the left or right dorsal thigh of the mice (n=10), respectively. The animals were monitored daily, and tumor volumes were measured every 2 days using a caliper slide rule. Tumor volumes were calculated as previously described 19, 20. 2 weeks after cancer cell implantation, mice were sacrificed, and tumor xenografts were dissected, weighed and fixed overnight in 4% paraformaldehyde, dehydrated, paraffin-embedded, sectioned and followed by H&E staining and BrdU staining. The animal experiments were carried out in strict accordance with the recommendations in the Guide for the Care and Use of Laboratory Animals of the Southern Medical University. The animal protocol was approved by the Committee on Ethics of Animal Experiments of the Southern Medical University. All surgery was performed under sodium pentobarbital anesthesia, and all efforts were made to minimize suffering of animals.

### Statistical analyses

The data were presented as mean ± SEM. Statistical analyses were conducted using the SPSS 22.0 software package and GraphPad Prism 8.0 software. A two-tailed Student's t test was used for comparisons of three independent groups. The χ2 test was used to analyze the association between clinicopathological characteristics and MST4 expression. Values are statistically significant at *P<0.05, **P<0.01 and ***P<0.001.

## Results

### MST4 is frequently down-regulated in HCC

**Figure 1:**
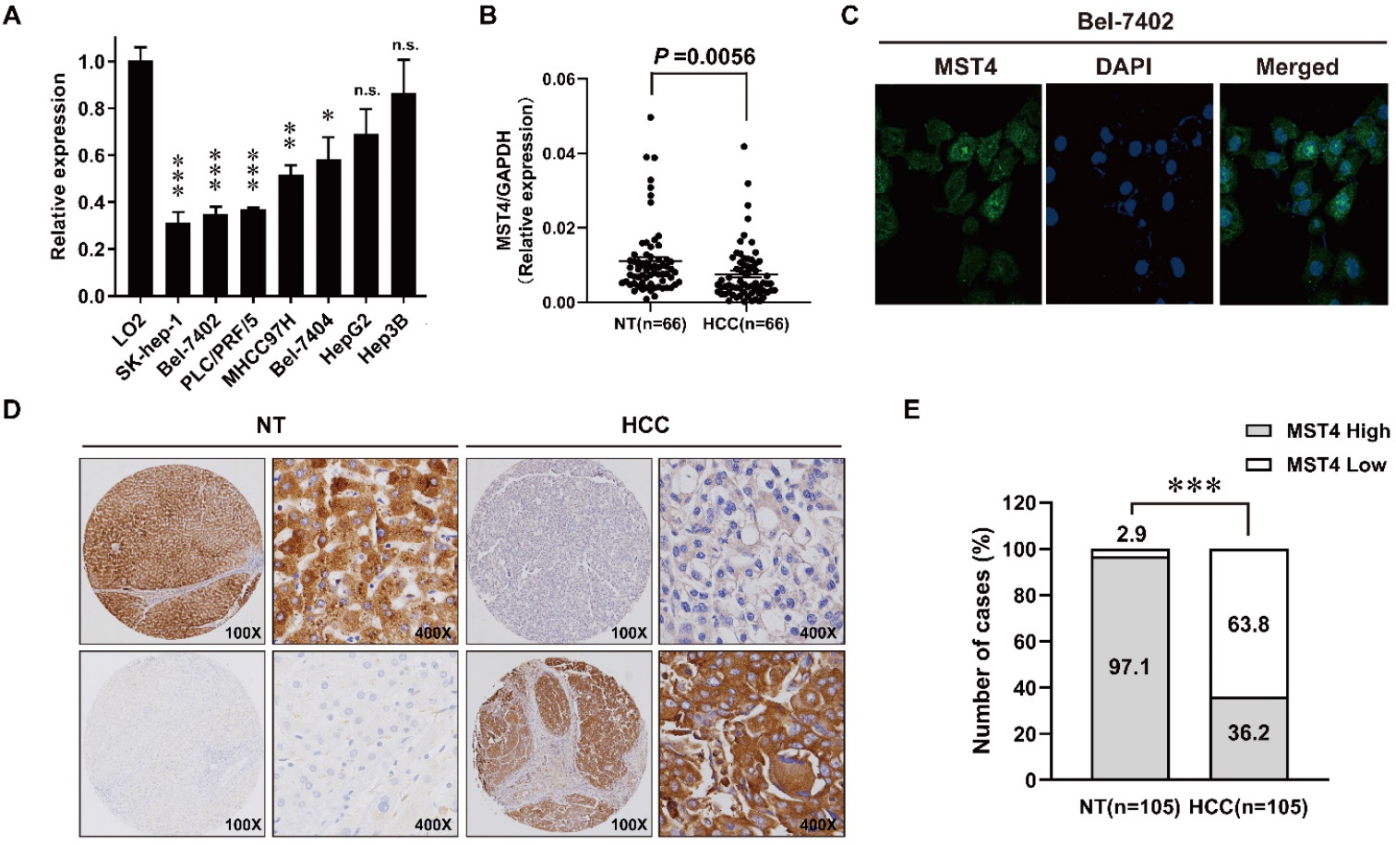
** Down-regulation of MST4 in hepatocellular carcinoma (HCC). (A)** MST4 mRNA expression in the indicated 7 HCC cell lines and normal human hepatic cell line LO2 detected by qRT-PCR. **(B)** MST4 mRNA expression in HCC and matched NT specimens from 66 HCC patients detected by qRT-PCR (P value shown was calculated by a paired Student's t test). **(C)** Representative images of MST4 protein localization in Bel-7402 cells by immunofluorescence (IF) staining. **(D)** Representative images showing MST4 protein weak expression in HCC and high expression in NT specimens examined by immunohistochemistry (IHC) staining. **(E)** The percentage of different MST4 IHC staining levels in the HCC and NT specimens. NT: adjacent non-tumor liver tissue.

We first examined the expression profiles of MST4 in seven human HCC cell lines and a human hepatic cell line LO2 using qRT-PCR. As shown in Fig. 1A, the expression of MST4 in most of the HCC cell lines tested is lower than that of LO2. Next, we detected the expression of MST4 in 66 pairs of human HCC and adjacent noncancer liver tissues at the mRNA level. Our data showed that the expression of MST4 was significantly lower in HCC tissues than their matched adjacent noncancer liver tissues (Fig. 1B). Subsequently, we performed immunofluorescence assay in Bel-7402 cell line. The results showed that MST4 protein was primarily located in the cytoplasm, and nuclear accumulation of MST4 was found in a small fraction of HCC cells (Fig. 1C). For MST4 IHC staining in HCC and adjacent noncancer liver tissues, immunoreactivity was mainly observed in the cytoplasm (Fig. 1D). We examined MST4 expression in 105 pairs of paraffin-embedded human HCC and adjacent noncancer liver tissues by IHC analysis. High expression of MST4 was detected in 102 cases of 105 adjacent noncancer liver tissues (97.1%), and that of MST4 in HCC tissues was 36.2% (38 cases) (Fig. 1D, E, Table S1), indicating that MST4 is frequently downregulated in human HCC clinical specimens.

### MST4 inhibits HCC proliferation *in vitro*

**Figure 2:**
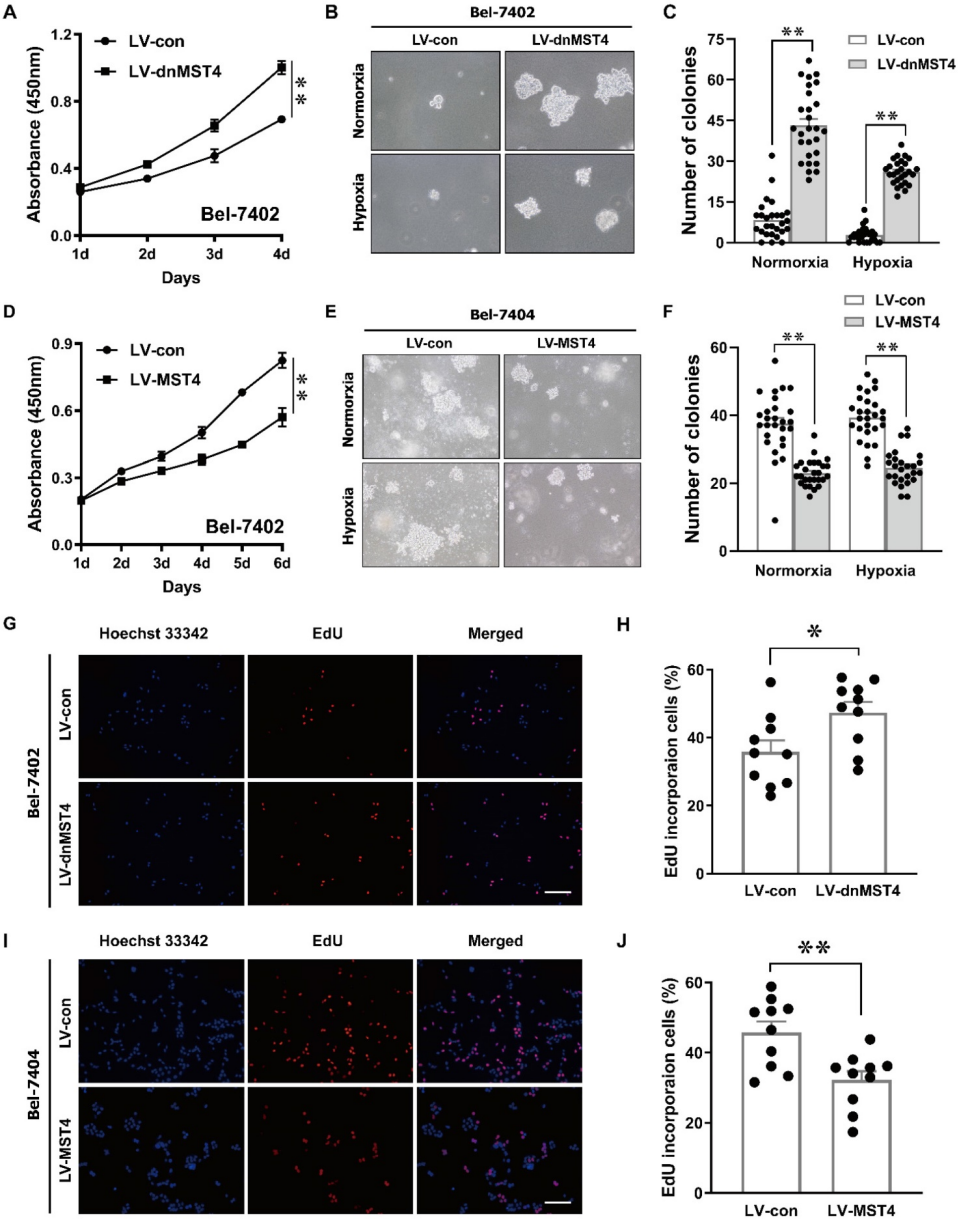
** MST4 negatively regulates HCC cell proliferation *in vitro*. (A)** Cell proliferation of dnMST4- and vector-expressing Bel-7402 cells was detected by CCK-8 assay (**P < 0.01, Student's t test). **(B-C)** The anchorage-independent growth of dnMST4- and vector-expressing Bel-7402 cells under normoxia and hypoxia was detected by soft agar assays, and the statistical results were shown in pane (C) (**P < 0.01). **(D)** Effects of MST4 overexpression on Bel-7402 cell proliferation were determined by CCK-8 assay (**P < 0.01, Student's t test). **(E-F)** The anchorage-independent growth of MST4-overexpressing and vector-expressing Bel-7404 cells under normoxia and hypoxia were interrogated by soft agar colony formation assays, and the statistical results were shown in pane (F) (**P < 0.01). **(G-H)** The percentages of proliferating cells in dnMST4- and vector-expressing Bel-7402 cells were determined by EdU incorporation assay, and the statistical results were shown in pane (H) (*P < 0.05). Scale bar, 500μm. **(I-J)** The percentages of proliferating cells in MST4-overexpressing and vector-expressing Bel-7404 cells were determined by EdU incorporation assay, and the statistical results were shown in pane (J) (**P < 0.01). Scale bar, 500μm.

In order to investigate the effect of MST4 functional inactivation on the proliferation of HCC cells, we stably introduced a dominant-negative mutant of MST4 (dnMST4) and control plasmids into Bel-7402 cells, respectively. The dominant-negative mutant can cause an altered gene product that antagonizes the wild-type MST4 alleles. CCK-8 assays revealed that dnMST4-expressing Bel-7402 cells grew more rapidly than control cells (Fig. 2A). Soft agar assays showed that the expression of dnMST4 significantly promoted the frequency of colony formation, indicating that functionally inactivation of MST4 can promote HCC cell growth in an anchorage-independent manner (Fig. 2B, C). To further confirm the suppressive function of MST4 on HCC cell proliferation, we ectopically overexpressed wild-type MST4 in Bel-7402 and Bel-7404 cells. CCK-8 assays were employed to evaluate the effects of MST4 overexpression on cell proliferation *in vitro*. As presented in Fig. 2D, MST4 remarkably decreased the proliferative capacity of Bel-7402 cells. Moreover, soft agar assays demonstrated that exogenous expression of MST4 in Bel-7404 cells substantially reduced the HCC cell ability of colony formation (Fig. 2E, F). Consistently, dnMST4 expression markedly increased the percentage of EdU incorporation into Bel-7402 cells (Fig. 2G, H), whereas overexpression of MST4 led to a significant decrease in EdU incorporation (Fig.2I, J). Collectively, these results suggest that MST4 negatively regulates *in vitro* HCC cell proliferation.

### MST4 suppresses tumor growth of HCC cells in nude mice

**Figure 3:**
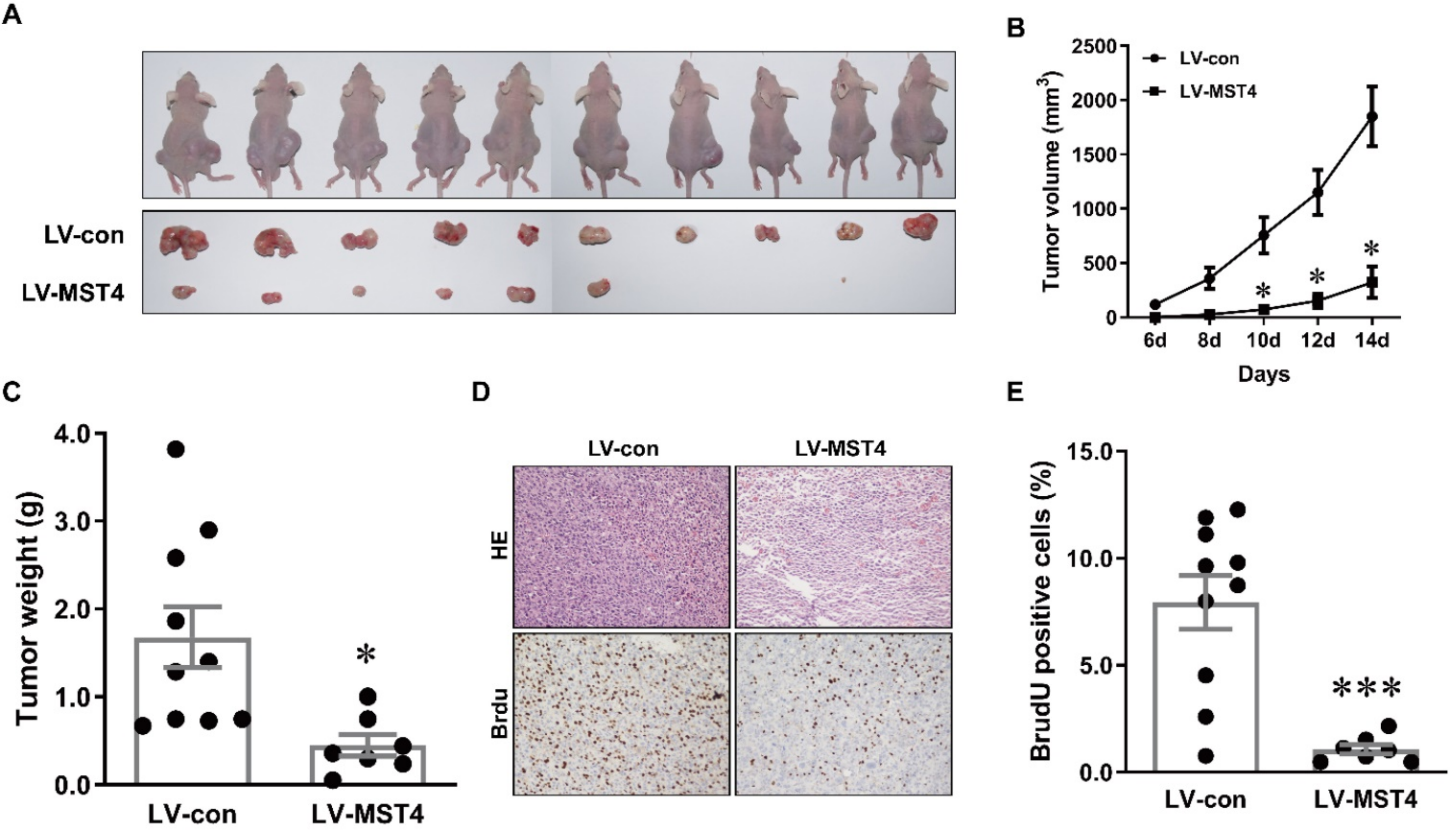
** MST4 inhibits HCC cell xenograft growth in nude mice. (A)** Representative pictures of xenograft tumors from subcutaneous injection of MST4-overexpressing and vector-expressing Bel-7404 cells into nude mice. **(B)** Tumor growth curves of MST4-overexpressing and vector-expressing Bel-7404 cells (*P < 0.05). **(C)** Tumor weight at 14th day after the inoculation of MST4-overexpressing and vector-expressing Bel-7404 cells in nude mice (*P < 0.05). **(D)** Representative images of HE and BrdU staining of xenograft tumors of MST4-overexpressing and vector-expressing Bel-7404 cells. **(E)** The percentage of BrdU-positive cells in tumor xenografts formed by MST4-overexpressing and vector-expressing Bel-7404 cells examined by IHC (***P < 0.001).

To further examine the anti-tumor effect of MST4 on HCC *in vivo*, a xenograft model was established by subcutaneously injecting MST4-overexpressing and vector-expressing Bel-7404 cells into the flanks of nude mice. Tumor growth was evaluated by measuring the tumor size every two days. Fourteen days after injection, tumor formation was observed in 7 out of 10 mice (for MST4-overexpressing cells) and 10 out of 10 mice (for vector-expressing cells) (Fig. 3A). The tumor size and volume induced by MST4-overexpressing cells were significantly smaller than those induced by vector-expressing cells (Fig. 3A, B). Tumor weights in the MST4 overexpression group were noticeably lower than those in the control group (Fig. 3C). Additionally, BrdU is an analog of thymine nucleoside, which can be easily incorporated into DNA during DNA synthesis and used for accurately monitoring cell proliferation. The immunohistochemical staining of BrdU in xenograft tumor tissues showed that the proportion of BrdU-positive cells in MST4 overexpression group was appreciably lower than that in the control group (Fig. 3D, E). In all, these findings demonstrated that MST4 suppresses *in vivo* tumor growth of HCC cells.

### MST4 inhibits the G1-S phase cell cycle transition of HCC cells

**Figure 4:**
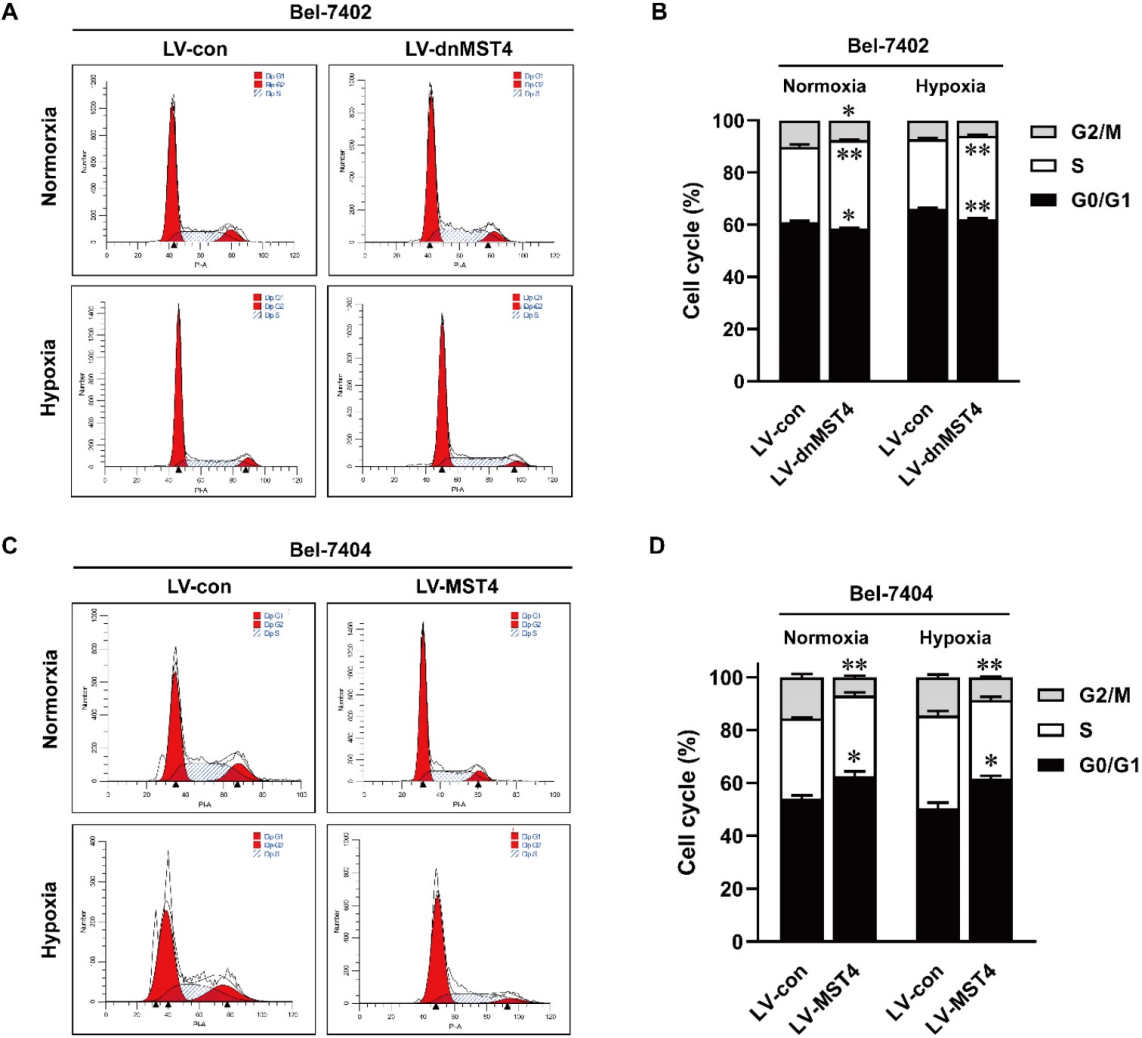
** The effects of MST4 on cell cycle of HCC cells. (A-B)** Cell cycle distributions in dnMST4- and vector-expressing Bel-7402 cells under normoxia and hypoxia were detected by PI staining in triplicate followed by flow cytometry, and the statistical results were shown in pane (B) (*P <0.05, **P <0.01). **(C-D)** Cell cycle distributions in MST4-overexpressing and vector-expressing Bel-7404 cells under normoxia and hypoxia were detected by PI staining in triplicate followed by flow cytometry, and the statistical results were shown in panel (D) (*P < 0.05, **P < 0.01).

To elucidate the underlying mechanism of MST4 for HCC cell growth inhibition, we analyzed the cell cycle by PI staining. As shown in Fig. 4A, B, dnMST4 expression in Bel-7402 cells decreased the proportion of G1 phase cells, while at the same time decreased G2/M phase cells and increased S phase cells. In contrast, MST4 overexpression in Bel-7404 cells showed a markedly G1 phase arrest. The increased proportion of G1 phase cells was found in MST4-overexpressing Bel-7404 cells, while decreased G2/M phase cells and unchanged S phase cells were observed (Fig. 4C, D). Taken together, these data showed that MST4 altered the cell cycle distribution of HCC cells, leading to a cell cycle arrest of G1 phase. These results suggest that the growth-suppressive effect of MST4 overexpression was linked to the G1 phase cell cycle arrest.

### MST4 suppresses HCC cell proliferation and induces G1 phase cell cycle arrest by inactivating PI3K/AKT signaling pathway

**Figure 5:**
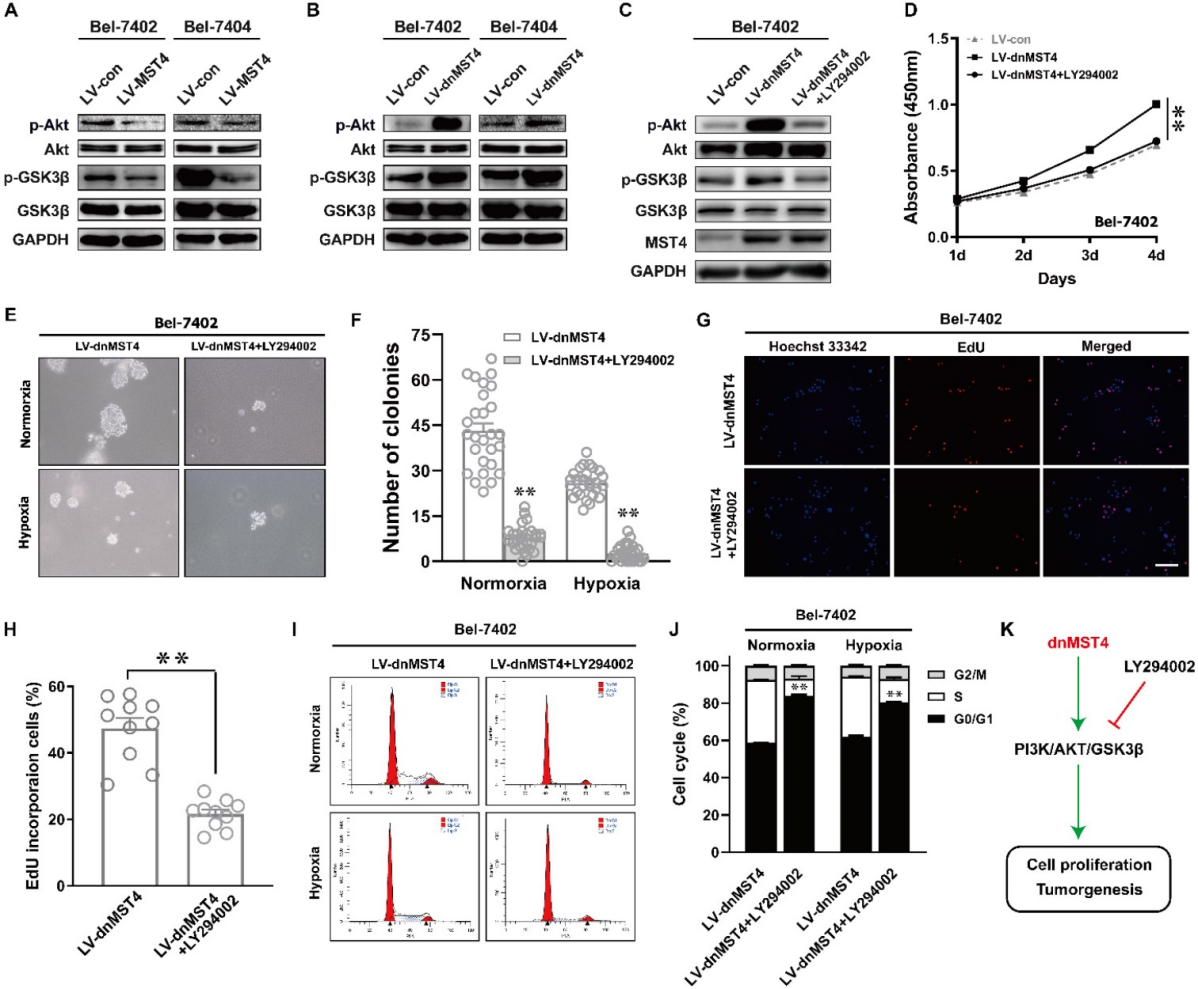
** MST4 regulates HCC cell proliferation and cell cycle by affecting PI3K/AKT pathway. (A)** Western blot analysis of AKT, p-AKT, GSK3β and p-GSK3β in MST4-overexpressing Bel-7402 and Bel-7404 cells. **(B)** Western blot analysis of AKT, p-AKT, GSK3β and p-GSK3β in dnMST4-expressing Bel-7402 and Bel-7404 cells. **(C)** Western blot analysis of AKT, p-AKT, GSK3β and p-GSK3β in dnMST4-expressing Bel-7402 cells and dnMST4-expressing Bel-7402 cells treated with LY294002 for 24 hours. **(D)** Cell proliferation of dnMST4-expressing Bel-7402 cells and dnMST4-expressing Bel-7402 cells treated with LY294002 detected by CCK-8 assay (**P < 0.01, Student's t test). **(E-F) The** anchorage-independent growth of dnMST4-expressing Bel-7402 cells and dnMST4-expressing Bel-7402 cells treated with LY294002 under normoxia and hypoxia was interrogated by soft agar assay, and the statistical results were shown in pane (F) (**P < 0.01). **(G-H)** The percentage of proliferating cells in dnMST4-expressing Bel-7402 cells and dnMST4-expressing Bel-7402 cells treated with LY294002 was determined by EdU incorporation assay, and the statistical results were shown in pane (H) (*P < 0.05). Scale bar, 500μm. **(I-J)** Cell cycle distributions in dnMST4-expressing Bel-7402 cells and dnMST4-expressing Bel-7402 cells treated with LY294002 under normoxia and hypoxia were detected by PI staining in triplicate followed by flow cytometry, and the statistical results were shown in pane (J) (**P < 0.01). **(K)** A schematic diagram of the signal pathway that MST4 inhibits HCC cell proliferation and induces cell cycle arrest via suppression of PI3K/AKT pathway.

The PI3K/AKT pathway plays an important role in regulating tumor cell proliferation and cell cycle 21, 22. Therefore, we evaluated the protein levels of AKT phosphorylation in MST4-overexpressing Bel-7402 and Bel-7404 cells. Compared with control cells, the phosphorylation levels of AKT in MST4-overexpressing cells were decreased, and there was no discrepancy in the total protein level of AKT between the two groups (Fig. 5A). As one of the downstream molecules of PI3K/AKT signaling pathway, GSK-3β has been reported to be phosphorylated and inactivated by AKT, thus promoting the G1-S phase cell cycle progression 23. MST4 overexpression reduced the phosphorylation levels of GSK-3β (Fig. 5A), which further confirms that the activity of PI3K/AKT pathway is suppressed. To verify those data, we further measured the phosphorylation levels of AKT and GSK-3β in dnMST4-expressing Bel-7402 and Bel-7404 cells. Our results showed that dnMST4 expression significantly increased the phosphorylation levels of AKT and GSK-3β, indicating that the activity of PI3K/AKT pathway is enhanced (Fig. 5B). Next, we employed the PI3K/AKT inhibitor (LY294002) to further determine the function of PI3K/AKT signaling pathway in MST4-mediated suppression of HCC cell proliferation. The dnMST4-expressing Bel-7402 cells were treated with LY294002, and then the phosphorylation levels of AKT and GSK-3β were detected. Our results showed that LY294002 treatment rescued the dnMST4-induced increased phosphorylation levels of AKT and GSK-3β (Fig. 5C). Subsequently, CCK8 assays revealed that the promoting effect of dnMST4 on the growth rate of Bel-7402 cells was severely compromised by LY294002 (Fig. 5D). This was confirmed by EdU incorporation assay. The proportion of EdU-positive cells in dnMST4-expressing Bel-7402 cells was significantly reduced in the presence of LY294002 (Fig. 5G, H). Additionally, soft agar assays showed that LY294002 markedly repressed the enforced ability of colony formation in dnMST4-expressing Bel-7402 cells (Fig. 5G, 5H). Finally, we analyzed PI staining in dnMST4-expressing Bel-7402 cells treated with LY294002. We found that LY294002 can significantly reverse the G1-S phase cell cycle progression induced by dnMST4 expression in Bel-7402 cells (Fig. 5I, J). Collectively, these results demonstrated that the inactivation of PI3K/AKT pathway is involved in the MST4-mediated suppression of HCC proliferation and G1 phase cell cycle arrest (Fig. 5K).

## Discussion

HCC is considered to be the second most lethal tumor, after pancreatic cancer, with a 5-year survival rate of 18% 1. Therefore, it is essential to explore the molecular mechanisms for the development of more effective therapeutic strategies of HCC. Based on tissues and human HCC cell lines, we explored the roles of MST4 in HCC. First, we found that the MST4 was remarkably downregulated in human HCC clinical tissues compared with adjacent noncancer liver tissues. Second, we showed that enforced expression of MST4 suppressed HCC cell proliferation and colony formation and induced G1 phase cell cycle arrest. In contrast, dnMST4 expression promoted HCC cell proliferation and colony formation, and enhanced the G1-S phase cell cycle transition. Third, we revealed that MST4 has a negative regulatory effect on PI3K/AKT signaling pathway, which is related to the anti-HCC effect of MST4. In addition, MST4 repressed the phosphorylation of GSK3β, which may be linked to its inhibition of cell cycle progression.

MST4 has been widely implicated in the progression of several types of human cancers including prostate cancer, breast cancer, glioblastoma, pancreatic cancer, and gastric cancer 11-14, 24. Evidence revealed that MST4 can facilitate epithelial to mesenchymal transition (EMT) and metastasis of HCC via activating ERK pathway 25. However, its actual role in HCC cell proliferation and underlying molecular mechanisms remains largely unknown. The potential for unlimited proliferation is a prominent feature of malignancies. According to our data, MST4 overexpression significantly inhibited cell proliferation and induced G1 phase cell cycle arrest of HCC cells. Moreover, the anchorage-independent growth ability of cancer cells is considered to be a hallmark of tumorigenesis 26, 27. Our results from soft agar assay showed that enforced expression of MST4 inhibited the anchorage-independent colony formation of HCC cells, indicating an inhibitory effect of MST4 on HCC tumorigenesis. Thus, these findings suggest a potential therapeutic value of MST4 in HCC treatment.

It is known that MST4 promoted the progression of prostate cancer and EMT and tumor metastasis of HCC and gastric cancer by activating p-ERK pathway 13, 25. For pancreatic cancer, MST4 interacted with MOB4 to form MST4-MOB4 complex, which antagonized MST1-MOB1 complex to positively regulate YAP activity, thus promoting the cell migration and proliferation 11. In glioblastoma, inhibition of MST4 inactivated ATG4B thus inhibits cancer cell autophagy and tumorigenicity 14. In addition, MST4 can also have a function within inflammatory responses by phosphorylation of TRAF6 28. PI3K/AKT signaling pathway is a classic pathway in tumorigenesis 29. Our results showed that MST4 overexpression dramatically decreased the phosphorylation levels of AKT but not altered the total level of AKT in HCC cells. Moreover, MST4 overexpression markedly downregulated the phosphorylated GSK3β at Ser9, which led to the G1 phase cell cycle arrest 30. These results suggest that MST4 might inhibit the proliferation of HCC cells by affecting the PI3K/AKT signaling pathway. In order to confirm the function of PI3K/AKT pathway in the inhibitory effect of MST4 on the proliferation of HCC cells, we inactivated MST4 in HCC cells by ectopic expression of a dominant-negative mutant of MST4 protein and intervened PI3K/AKT pathway with PI3K inhibitor LY294002. Our results showed that dnMST4 could facilitate the proliferation and anchorage-independent growth of HCC cells, while the growth-promoting effect of dnMST4 was reversed by LY294002. In addition, we also observed that dnMST4 promoted the G1-S phase cell cycle transition of Bel-7402 cells, and this effect was recused in the presence of LY294002. These findings suggest an essential role for PI3K/AKT pathway in the inhibitory effects of MST4 on HCC cell proliferation and cell cycle progression.

The unlimited proliferation of cancer cells is usually caused by cell cycle disorders 31. Unlike normal cells, many cancer cells do not respond to growth inhibition signals, which are associated with components that control the cell cycle 32. Many carcinogenic factors play an important role in the regulation of cell cycle, especially in the cell transition of G1 to S phase 33. A great deal of evidence supports a key role of PI3K pathway in the progression of cell cycle. Its activity in G1 phase is necessary for subsequent initiation of DNA synthesis 34. In our study, we found that MST4 overexpression blocked HCC cells in G1 phase, accompanied by a significant decrease in cell proliferation compared with the control group. The experimental data *in vivo* indicated that MST4 can exhibit an inhibitory effect on tumor growth. Western blot analysis provided molecular evidence to support these observations, that is, the overexpression of MST4 reduced the phosphorylation levels of AKT and GSK3β, while dnMST4 expression led to an elevated level. GSK3β is a serine/threonine phosphate kinase and a pivotal downstream target protein of PI3K/AKT pathway 35. Its activity is silenced by AKT when it is phosphorylated at Ser9 36. Suppression of GSK-3β can stabilize β-catenin which leads to the upregulation of c-Myc, survivin, cyclin D1 and cyclin E, thereby overcoming G0/G1 phase cell cycle arrest and promoting cell proliferation and tumorigenesis 36, 37. Meanwhile, PI3K/AKT/GSK3β pathway can also modulate cell cycle by regulating p21 protein phosphorylation 38. Therefore, it is speculated that MST4 may suppress HCC cell proliferation and tumor growth via inhibiting cell cycle progression by inactivating PI3K/AKT/GSK3β pathway.

In summary, we prove for the first time that MST4 is generally downregulated in HCC tissues. Our study unravels an important role of MST4 in the suppression of HCC cell proliferation via blocking cell cycle progression, by a mechanism that involves the inactivation of PI3K/AKT signaling pathway. It is proposed that the specific functional target of MST4 in this pathway may need to be further studied. Overall, the findings of our study provide new insights into the clinical value and the underlying molecular mechanisms of MST4 in HCC.

## Supplementary Material

Supplementary table S1.

## Abbreviations

MST4: mammalian STE20-like protein 4
HCC: hepatocellular carcinoma
NT: adjacent noncancer liver tissues
IHC: immunohistochemistry
qRT-PCR: quantitative real-time PCR
