# Supplementary material for: MST4 inhibits human hepatocellular carcinoma cell proliferation and induces cell cycle arrest via suppression of PI3K/AKT pathway
Source: J Cancer. 2020 Jun 28;11(17):5106–17. doi: 10.7150/jca.45822 (PMC7378920; doi:10.7150/jca.45822)
Supplement: Supplementary file 1 — Supplementary table S1. [file jcav11p5106s1.pdf]

Table S1. Expression of MST4 in 105 Hepatocellular carcinoma (HCC) tissues and paired adjacent noncancer liver tissues (NT)

Variables	*n*	MST4 expression		χ^2	*P*
		Low (*n*, %)	High (*n*, %)		
NT	105	3 (2.9)	102 (97.1)	85.05	<0.0001
HCC	105	67 (63.8)	38 (36.2)		
